# Comparison of individual component deletions in a glucose-specific phosphotransferase system revealed their different applications

**DOI:** 10.1038/srep13200

**Published:** 2015-08-19

**Authors:** Quanfeng Liang, Fengyu Zhang, Yikui Li, Xu Zhang, Jiaojiao Li, Peng Yang, Qingsheng Qi

**Affiliations:** 1 State Key Laboratory of Microbial Technology, Shandong University, Jinan 250100, P. R. China

## Abstract

The phosphoenolpyruvate-dependent glucose-specific phosphotransferase system (PTS^Glc^) is the main glucose uptake pathway in *Escherichia coli* that affects both substrate assimilation and metabolism leading to the product formation. In this study, the effect of single PTS^Glc^ mutation on cell growth and substrate consumption was investigated by knocking out the genes involved in the phosphotransfer cascade of the PTS^Glc^. In addition, the distribution of the metabolites of mutants was analyzed. Each mutant was confirmed to have different adaptability in the presence of both glucose and xylose with different ratios, and a substrate mixture with high xylose content can be completely consumed in short time when the *ptsI* mutant is employed. Finally, *ptsH* deletion was for the first time applied for succinate production due to its well performance under anaerobic condition. Strain YL104H, in which *ptsH* was deleted, exhibited considerably increased succinate yield under both aerobic and anaerobic conditions. The succinate titer and overall productivity reached 511.11 mM and 1.01 g/L/h after 60 h during the whole-phase fermentation in a mineral salt medium. The present results demonstrated the glucose and xylose co-utilization efficiency and the product yield and productivity can be significantly improved if a suitable PTS^Glc^ deletion mutant was selected.

The key factors for developing bio-based industrial chemicals are increasing the substrate utilization and product yield. In *Escherichia coli*, the phosphoenolpyruvate (PEP)-dependent glucose-specific phosphotransferase system (PTS^Glc^) is the most efficient system for transporting glucose. The activity of the PTS^Glc^ has a vital effect on the carbon flux distribution and plays a key role in carbon catabolite repression (CCR).

In *E. coli,* several systems can transport sugar into the cytoplasm^1^,^2^. Among such systems, the PEP: sugar phosphotransferase system (PTS), is involved in PEP-dependent sugar transportation, whereas the PTS^Glc^ is the main glucose uptake and phosphorylation pathway, sharing two common phosphotransfer steps with other PTSs (Fig. 1). The PTS^Glc^ is composed of the soluble non-sugar-specific protein components Enzyme I (EI, *ptsI*) and histidine protein (HPr, *ptsH*), soluble glucose-specific enzyme IIA^Glc^ (*crr*), and membrane-integral glucose-specific permease IICB^Glc^ (*ptsG*) (Fig. 1). In *E. coli* K-12, the *ptsH*, *ptsI*, and *crr* genes are localized in the same operon; however, the *crr* gene has its own promoter^3^,^4^. Another key gene of the PTS^Glc^, *ptsG*, is located far from the *ptsHI*-*crr* operon^5^.

The PTS^Glc^ is a carbohydrate transport system and plays a crucial role in the global signaling system, called CCR, that controls the preferential consumption of glucose over other carbon sources; this process causes the sequential utilization of mixed carbohydrates and limits the application of low-cost lignocellulosic hydrolysates. In *E. coli*, CCR is caused by the modulation of the IIA^Glc^ phosphorylation status in the PTS^Glc^ 6. Numerous studies have used approaches including inactivating the PTS^Glc^ components for eliminating CCR in *E. coli*^7^,^8^. The *ptsG* gene has been frequently used as the first target because it separates from the other three genes and it encodes a component that locates the final step in the phosphotransfer cascade of the PTS^Glc^ (Fig. 1). Deleting the *ptsG* gene in *E. coli* reduced the specific growth rate to approximately 85% of that of the parent strain^9^ and resulted in the simultaneous assimilation of glucose and xylose^10^,^11^,^12^. Deleting another target in the PTS^Glc^, the entire *ptsHI-crr* operon, blocked the two common steps involved in the PEP: carbohydrate phosphotransfer cascades and resulted in a limited capacity for transporting glucose (Fig. 1)^13^,^14^. Another study reported that the *ptsHI-crr* mutant could partially diminish glucose repression on xylose^15^.

The PTS^Glc^ has a considerable effect on the carbon flux and distribution in central carbon metabolism^1^,^16^,^17^. PEP is a precursor in several biosynthetic pathways and participates directly in energy-generating reactions. Because 50% of the intermediate PEP produced from the catabolism of transported glucose is used as a phosphate donor for phosphorylating translocated glucose^7^,^18^, decoupling glucose transportation from PEP-dependent phosphorylation by deleting any gene in the PTS^Glc^ can save more PEP molecules for other metabolic pathways^7^,^13^, for example, the formation of succinate via reductive tricarboxylic acid cycles. Therefore, deleting the *ptsG* gene in an engineered *E. coli* resulted in a 22.5% increase in succinate production compared with that of the parent strain because of the decoupling of PEP-dependent glucose transportation and phosphorylation^19^. In addition, the disruption of the PTS^Glc^ reduced metabolic overflow, which is caused by combining the glucose uptake by the PTS and glucose catabolism through the Embden-Meyerhof-Parnas pathway, resulting in excess acetyl-CoA (acetate). Previous studies proved that deleting the *ptsG* gene reduced acetate secretion significantly^11^,^20^,^21^. A similar result was obtained in a strain with a *ptsHI-crr* operon deletion^13^. Meanwhile, deleting the *ptsHI-crr* operon in the PTS^Glc^ completely blocked PTS transfer and resulted in a limited capacity for transporting and phosphorylating glucose^22^.

In general, the PTS^Glc^ is a multicomponent system in which the cascade of phosphotransfer reactions, ratios of phosphorylated to non-phosphorylated forms, kinetics of the system, and response of the system to changes of metabolite concentrations are complex^19^,^23^. No study has reported a systematic analysis of the effect of deleting each component on substrate consumption, metabolite distribution, and cell growth. Furthermore, no study has analyzed the differential application of individual single gene knockout mutants. Obtaining more information on these key topics can lead to highly effective metabolism mediations and, finally, enhance the biomass co-utilization efficiency and product yield. In the present study, we analyzed the physiological changes in PTS^Glc^ mutants with individual gene deletions in *E. coli* and investigated the application of different PTS^Glc^ component deletions in glucose and xylose co-utilization efficiency and high-yield succinate production.

## Results and Discussion

### Disruption of different components of the PTS^glc^ cascade resulted in various levels of glucose consumption and cell growth

In *E. coli*, the assimilation of glucose via the PTS^Glc^ entails a series of cascade steps that involve four components: EI, HPr, IIA^Glc^, and IICB^Glc^. To investigate the effect of the inactivation of each component on substrate consumption and cell growth, we deleted the corresponding genes in the PTS^Glc^ of wild-type *E. coli* W3110, generating *E. coli* W3110I, W3110H, W3110C, and W3110G (Table 1). Cultivating these mutants in the presence of substrate glucose resulted in reduced glucose consumption and retarded growth under aerobic condition (Fig. 2a,b). Among them, W3110I demonstrated the longest lag phase (40 h). Moreover, W3110C showed the shortest lag phase of 4 h, similar to that of the wild-type strain W3110. Although the glucose consumption rates of W3110 and mutant are similar, these strains showed various lag period. In contrast to the wild-type strain, the lag times of W3110C, W3110G, W3110H and W3110I are 4, 15, 16, 38 h, respectively (Fig. 2b). However, the final biomasses of each mutant, W3110I, W3110H, W3110C, and W3110G, were 45.42%, 30.51%, 28.47%, and 12.88% higher than that of the wild-type strain W3110, respectively (Fig. 2a). Under anaerobic conditions, the growth of W3310 and mutants are very weak, however, they displayed similar sequence in growth lag times with those under aerobic condition (Fig. 2c).

**Figure 1:**
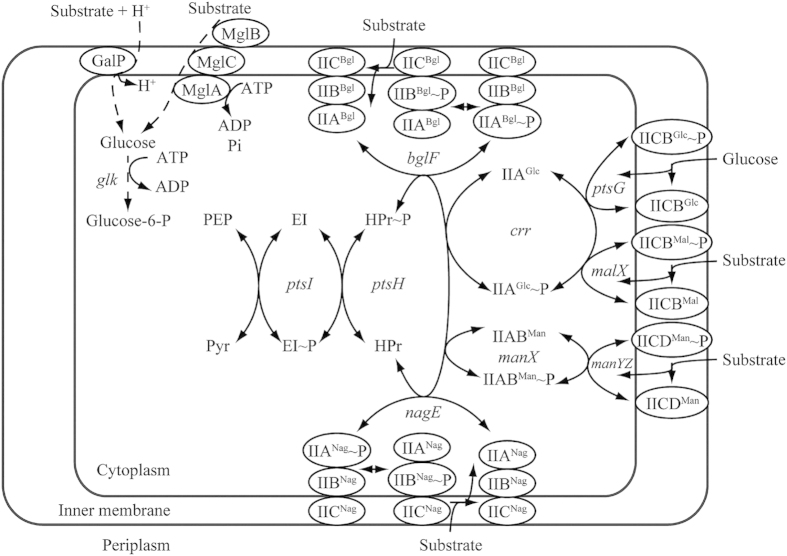
Transport systems capable of internalizing glucose in *E. coli*. The PTS^Glc^ is composed of the soluble non-sugar-specific protein component Enzyme I (EI, *ptsI*) and phosphohistidine carrier protein (HPr, *ptsH*), soluble glucose-specific enzyme IIA^Glc^ (*crr*), and membrane-integral glucose-specific permease IICB^Glc^ (*ptsG*). Other PTSs, specific for maltose, mannose, β-glucoside, and N-acetylglucosamine, can also transport glucose. The non-PTSs include the galactose: H^+^ symporter and galactose ABC transporter.

**Figure 2:**
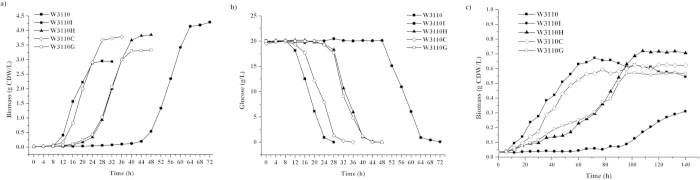
(**a**) Strain growth, and (**b**) glucose consumption under aerobic condition, as well as (**c**) strain growth under anaerobic condition.

The differences in the lag phase of each mutant may be attributed to the different induction and capacity of the alternative PTS or/and transport system for glucose assimilation after the blockage of the PTS^Glc^ at different points. *E. coli* contains several transport systems that can assimilate glucose, and such systems include PTS^Glc^, PTS^Mal^, and PTS^Man^ (Fig. 1). EI and HPr are the common transporter proteins in all PTSs; therefore, inactivating EI (strain W3110I) and HPr (strain W3110H) should entail recruiting a non-PTS transport system such as the galactose permease system, which may require a relatively long lag phase for induction. The experimental results support this assumption (Fig. 2a). The shorter lag time of growth and glucose consumption of W3110H, relative to those of W3110I, indicated that the protein EI, which plays a vital role in physiological signaling^24^,^25^, is more sensitive to glucose induction. Another reason is that EI autophosphorylation and the subsequent phosphotransfer reaction are the limiting steps in the phosphotransfer cascade of the PTS^Glc^ 17, whereas IIA^Glc^ and IICB^Glc^ are glucose-specific components; their inactivation mutants W3110C and W3110G could recruit both PTSs (for example, PTS^Mal^ and PTS^Man^) and non-PTSs for glucose internalization. Therefore, the growth lag phase of W3110I was relatively long. The glucose consumption and growth profiles of W3110C were similar to those of the wild-type W3110.

### Deletions of individual PTS^Glc^ components exhibited changed product distributions

**Figure 3:**
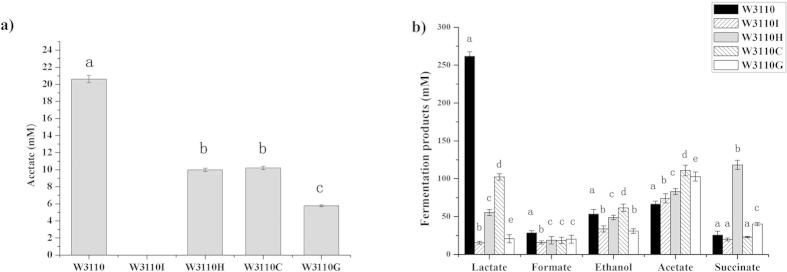
(**a**) Acetate secretion of *E. coli* W3110 and four PTS^Glc^ mutants in the mineral medium supplied with 20 g l^−1^ glucose under aerobic conditions. The pH was not controlled. (**b**) Distribution of the metabolites of *E. coli* W3110 and four PTS^Glc^ mutants under anaerobic conditions. A *P* value of <0.05 was considered statistically significant. Different letters above the bars indicate significant differences (*P *< 0.05).

Different substrate assimilation rates may cause the accumulation of different metabolites. Acetate is usually accumulated because of the metabolic overflow in *E. coli*, and it is an undesirable byproduct in metabolic engineering and protein overexpression^26^. All the PTS^Glc^ mutants showed an obvious reduction in the concentration of acetate. The maximum acetate concentrations of W3310, W3110H, W3110C, and W3110G in the culture were 20.61, 9.97, 10.20, and 5.76 mM, respectively, and no acetate was detected in W3110I (Fig. 3a). These results were correlated to the longer lag phases, even when the total fermentation time was 70 h. This indicated that the metabolic overflow in these mutants was diminished because of the inactivation of the PTS^Glc^ components. This finding can also be confirmed by the observation that the concentration of the final biomass in all four mutants was greater than that of the wild-type strain W3110 (Fig. 2a).

When we analyzed the acetate secretion under anaerobic conditions, we found that the acetate secretion in W3310I, W3110H, W3110C, and W3110G increased by 13.02%, 26.62%, 69.21%, and 56.9% (Fig. 3b), respectively, compared with that of the wild-type strain W3110. Although there were no significant differences in the formate, acetate and ethanol secretion between the mutants and the wild-type strain, the lactate secretion in mutants decreased significantly. The most noteworthy observation under anaerobic conditions was the high accumulation of succinate in the W3110H mutant. The succinate product was the dominant fermentation product in W3110H, and the concentration of succinate acid in this mutant reached 118.21 mM, 4.6-fold higher than that in the wild-type strain W3310.

### Different co-utilization phenomena of glucose and xylose in PTS^Glc^ component deletions

**Figure 4:**
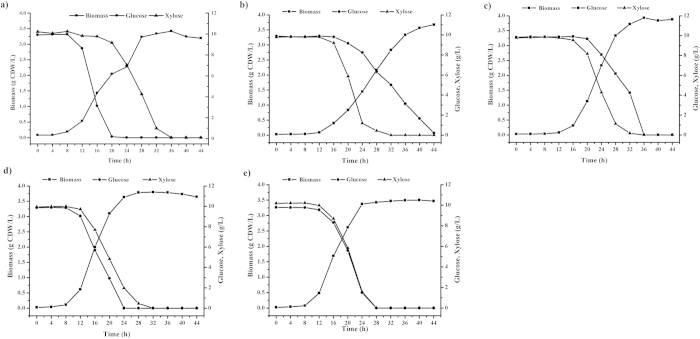
Growth and metabolism profiles of the mutants in a mixture of glucose and xylose at an identical mass concentration. (**a**) W3110, (**b**) W3110I, (**c**) W3110H, (**d**) W3110C, and (**e**) W3110G. The strains were grown in a mineral medium supplemented with 10 g/L glucose and 10 g/L xylose as the carbon sources.

Glucose is the preferred carbon and energy source for cells and represses the assimilation and metabolism of other sugars, including xylose. In our previous studies, we confirmed that inactivation of the key component in the PTS^Glc^, IICB^Glc^ (*ptsG*), can enable reducing the inhibitory effect of glucose on other sugars^10^,^11^. To investigate whether the inactivation of other PTS^Glc^ components results in similar effects, we cultivated the mutants in the medium containing both glucose and xylose at identical mass concentrations. The results indicated that all the PTS^Glc^ mutants used glucose and xylose simultaneously. However, they exhibited different co-utilization phenomena (Fig. 4 and Table 2). The wild-type W3110 strain preferred using glucose as its carbon source and then metabolized xylose when glucose was exhausted (Fig. 4a). The W3110C mutant showed a high glucose consumption rate and consumed glucose and xylose at a rate of 0.72 and 0.40 g/g CDW/h, respectively. The W3110G mutant consumed glucose and xylose simultaneously at almost the same rate (i.e., 0.37 and 0.38 g/g CDW/h, respectively; Fig. 4e and Table 2). The W3110I and W3110H mutants showed higher consumption rates for xylose than for glucose. Specifically, the W3110I mutant had a xylose consumption rate of 0.88 g/g CDW/h and glucose consumption rate of 0.24 g/g CDW/h (Fig. 4b,c and Table 2), which may be suitable for a substrate mixture with high xylose content. We also investigated the performance of each mutant in different mass ratios of glucose and xylose (2:3 and 3:2). We discovered that the co-utilization characteristics of glucose and xylose in W3110I, W3110H, W3110C, and W3110G did not depend on the mass ratio of the two carbon sources (Table S2).

Although the co-utilization phenomena of glucose and xylose were different, all the mutants accumulated approximately the same final biomass (Fig. 4). We assumed that the disruption of the components at different steps in the PTS^Glc^ affected the phosphorylation status of each component, resulting in different growth rates and changing the hierarchical structure of carbon source utilization in the presence of both glucose and xylose.

### Application of *ptsI*mutant in substrate mixture with high xylose content

**Figure 5:**
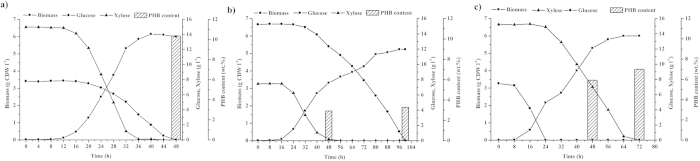
Batch cultivation of W3110I/p5TPHB in mixtures of glucose and xylose with different mass concentration ratios. (**a**) Ratio of glucose to xylose of 1:2, (**b**) ratio of glucose to xylose of 2:1; (**c**) Batch cultivation of W3110/p5TPHB in mixtures of glucose and xylose with concentration ratio of 1:2.

Because W3110I showed a higher consumption rate of xylose than that of glucose, we investigated its performance in a substrate mixture with a high xylose concentration by using *in vivo* PHB biosynthesis as an indicator. When glucose and xylose were introduced in a mixture with a mass concentration ratio of 1:2, the W3310I harboring the PHB biosynthesis pathway of PHB from *Ralstonia eutropha* H16 (W3110I/p5TPHB) completely consumed glucose and xylose in 48 h and accumulated 10.36% (w/w) of PHB (Fig. 5a). The overall productivity of PHB was 13.27 mg/L/h. However, when glucose and xylose were introduced in a mixture with a mass concentration ratio of 2:1, xylose was still completely consumed in 50 h, whereas glucose was completely consumed in 100 h (Fig. 5b). The final PHB content was only 3.28% (w/w) and the PHB productivity was 1.72 mg/L/h, seven fold lower than that in the substrate mixture with a ratio of 1:2 (glucose:xylose). These results clearly demonstrated that the W3110I utilized xylose faster than glucose and is more suitable for the substrate mixture with a high xylose concentration. Meanwhile, wild type strain W3310 harboring the PHB biosynthesis pathway of PHB (W3110/p5TPHB) only accumulated 5.92% (w/w) of PHB in 48 h in the substrate mixture with same ratio (Fig. 5c).

Glucose and xylose are the two major sugars, accounting for more than 90% of the total sugars in lignocellulosic hydrolysates^27^,^28^,^29^. The relative proportion of glucose and xylose in lignocellulose varies widely among sources^30^. For example, glucose and xylose account for 60% and 30% of the total sugar content in sugarcane bagasse hydrolysate, whereas they account for 10% and 80% of the sugar content in corn stalk hydrolysate^29^. Therefore, the complete and efficient utilization of these biomass requires the presence of microorganisms with different capabilities in glucose and xylose co-utilization. Deleting different components of the PTS^Glc^ adequately meets this demand, and mutants with such deletions can be used as hosts in a medium with different glucose and xylose concentrations.

### Application of *ptsH* mutant in high-yield succinate production from glucose

Because the anaerobic metabolite distribution analysis indicated that W3110H accumulated a high amount of succinate (Fig. 3b), we assumed that deleting *ptsH* may facilitate the succinate formation. Therefore, we constructed a new succinate producing strain, YL104H, based on our previously constructed strain YL104^31^. Dual-phase fermentation, which included an aerobic growth phase and anaerobic accumulation phase, indicated that the growth of YL104H was more favorable than that of YL104, and the final biomass of these strains was 2.73 and 1.93 g CDW/L, respectively. After 70 h cultivation, YL104H consumed 34.68 g/L glucose and produced 230.68 mM succinate, whereas YL104 consumed 28.49 g/L glucose and produced 162.62 mM succinate (Table 3).

Remarkably, a higher succinic acid yield was obtained after the *ptsH* gene was inactivated. The yields of succinic acid in YL104H under aerobic and anaerobic conditions were 0.90 and 1.65 mol/mol, respectively. This is the first reported strain that can produce succinate with a particularly high yield under both aerobic and anaerobic conditions. The yields of succinic acid in YL104 were 0.78 and 1.46 mol/mol, respectively (Table 3).

**Figure 6:**
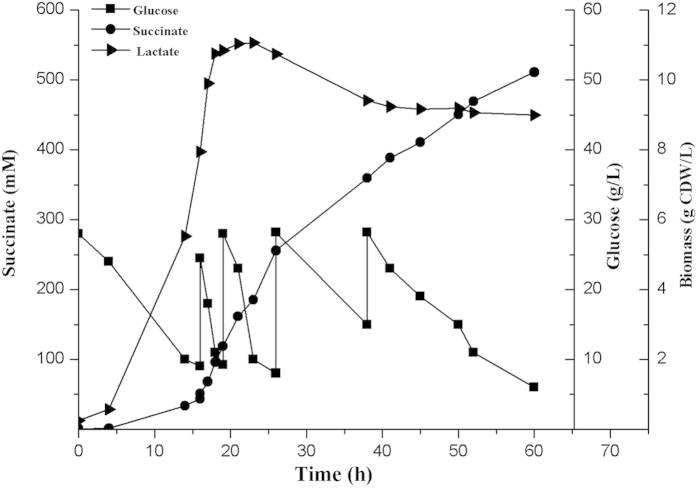
Whole-phase fed-batch fermentation of YL104H. The agitation rate was maintained at 500 rpm. The air flow was initially 1 vvm; after 12 h, it was dropped to 0.1 vvm; then after 32 h, the air flow was dropped to 0 vvm until fermentation ended.

The production of succinate of YL104H was further investigated using the whole-phase fermentation strategy (Fig. 6). After 60 h of cultivation in the mineral medium, the final succinate titer reached 501.11 mM. The overall volumetric productivity of succinate was 8.52 mol/(L·h), which corresponds to 1.01 g/L/h. The concentration of the byproducts was extremely low.

We assumed that the high PEP pool, which was induced by the inactivation of *ptsH*, can further increase the succinate production. When *E. coli* is grown in a minimal medium containing glucose as the carbon source, the PTS consumes 50% of the available PEP, whereas the reactions catalyzed by enzymes, such as PEP carboxylase, pyruvate kinases, consume the remaining PEP^7^,^18^. Several strategies have been developed for increasing the availability of PEP and redirecting it to biosynthetic pathways to achieve an increase in the production and yield of desired products^31^,^32^. The *ptsG* gene in PTS^Glc^ is usually inactivated for increasing the PEP pool. However, the inactivation of *ptsH* completely blocks the conversion of PEP to pyruvate and should further increase the PEP pool (Fig. 1). In the meantime, the pyruvate-forming flux was reduced, which is crucial for enhancing succinate production in the anaerobic fermentation of *E. coli*^33^.

Optimizing the yield, concentration, and productivity of commercially viable strains is imperative. Among these metrics, yield has the high priority^34^. Numerous researchers have attempted to achieve a high succinate yield under aerobic or anaerobic conditions by using engineered *E. coli*^19^,^35^,^36^,^37^,^38^,^39^. However, high-yield succinate mostly was obtained in a complex nutrient medium^19^,^35^,^36^,^37^. Among the strains constructed in the current study, the engineered *E. coli* HL27659K (pKK313) produced succinate at a yield of 0.95 mol/mol under aerobic conditions in a complex nutrient medium^19^. Under anaerobic conditions in minimal mediums, the succinate yield reached 1.5 mol/mol^38^ and 1.63 mol/mol^39^, respectively. In this study, the succinate yield of YL104H was relatively high under both aerobic and anaerobic conditions in the minimal medium, which resulted in a high productivity, in comparison to YL104. In the previous study, *ptsG* or *ptsI* in the PTS^Glc^ was usually used as the deletion target for improving the PEP pool and succinate production^31^,^40^. We proved for the first time in this study that *ptsH* is the most efficient candidate upon analyzing the anaerobic performance of each PTS^Glc^ mutant.

## Methods

### Bacterial strains

Table 1 and Table S1 list the strains and oligonucleotides used in this study, respectively. Four single-gene-deficient mutants were obtained from the parent strain *E. coli* K-12 W3110 through gene knockout by using a one-step inactivation method^41^. The primers *ptsI*-pKD4-F and *ptsI*-pKD4-R, *ptsH*-pKD4-F and *ptsH*-pKD4-R, *crr*-pKD4-F and *crr*-pKD4-R, and *ptsG*-pKD3-F and *ptsG*-pKD3-R in addition to the template plasmids pKD4^41^ for *ptsI*, *ptsH* and *crr* knockouts and pKD3^41^ for *ptsG* knockout were used to obtain linear DNA fragments with appropriate antibiotic-resistant gene cassettes flanked by FLP recognition target sites and 39-bp homologous arms; the obtained fragments were used for constructing the four single PTS^Glc^ gene mutants. After DNA purification, a polymerase chain reaction (PCR) product was electroporated into *E. coli* K-12 W3110 cells harboring the helper plasmid pKD46^41^ expressing Red recombinase enzymes. Positive clones were selected using appropriate antibiotics and confirmed through PCR. After the helper plasmid pKD46 was eliminated, the resistance gene was removed from the chromosome containing the helper plasmid pCP20^42^ expressing FLP recombinase. YL104H was also constructed using the same method with the *ptsH* knockout described previously, except *E. coli* K-12 YL104 was used as the parent strain. Regarding the production of polyhydroxybutyrate (PHB), a low-copy-number plasmid p5TPHB^43^, which contained an exogenous PHB biosynthesis pathway (constitutive expression of *phaCAB* operon) from *Ralstonia eutropha* H16, was introduced into W3110I, thus generating the PHB producer W3110I/p5TPHB. Spectinomycin was added to provide selective pressure during fermentation at a concentration of 25 μg/mL.

### Culture medium and growth conditions

During the construction of the single-gene-deficient mutants, cultures were grown aerobically at 30 °C, 37 °C, or 42 °C in an SOB medium (20 g/L tryptone, 5 g/L yeast extract, 0.5 g/L NaCl, 2.5 mM KCl, and 10 mM MgCl_2_) containing appropriate antibiotics (100 μg/mL ampicillin, 25 μg/mL kanamycin, and 17 μg/mL chloromycetin). The seeds were cultured in Luria-Bertani (LB) medium (10 g/L tryptone, 5 g/L yeast extract, and 10 g/L NaCl, pH 7.2). Furthermore, a mineral AM1 medium^44^ supplemented with glucose or both glucose and xylose was used for comparing the growth and metabolism profile of the four single PTS^Glc^ mutants and their parent strain.

A single clone of each single PTS^Glc^ gene mutant and the parent strain was cultured in a 300-mL Erlenmeyer flask containing 50 mL of LB medium at 37 °C and 250 rpm for 12 h. Next, the 500 μL seed culture was harvested through centrifugation (12 000 rpm for 2 min) and the cells were washed twice in the AM1 medium and inoculated in 50 mL of the mineral AM1 medium with the indicated carbon source at 37 °C and 250 rpm. Samples were collected at an appropriate interval.

Regarding the “dual-phase” fermentation in multiple minifermentor systems (Infors HT, Switzerland), a single clone was inoculated in the 300-mL Erlenmeyer flask containing 50 mL of LB medium at 37 °C and 250 rpm for 12 h. Next, 4 mL of the culture was inoculated into 80 mL of the mineral AM1 medium supplemented with 1 g/L yeast extract and 30 g/L glucose and cultivated for 21 h by using the same conditions. The seed culture (10% v/v) was then transferred into 800 mL of the mineral AM1 medium supplemented with 30 g/L glucose for batch fermentation. The pH, temperature, air flow rate, and agitation were maintained at 6.5, 37 °C, 1 vvm, and 250 rpm, respectively. The total fermentation time was 70 h, and it involved a 30-h aerobic phase (with O_2_) and 40-h anaerobic phase (with CO_2_). Samples were collected at an interval of 6 or 8 h.

Regarding the “whole-phase” fermentation in a 1-L jar, the agitation rate was maintained at 500 rpm. The initial air flow rate was 1 vvm and was subsequently reduced to 0.1 vvm when the strain was grown into the late exponential phase; after 32 h, the air flow rate was reduced to 0 vvm. Samples were collected at an appropriate interval.

### Analysis of substrates and products

Biomass was defined as the amount of cells (cell dry weight, CDW) per liter of culture broth. The CDW was calculated by measuring the optical density value at 550 nm (1 OD550 = 0.33 g CDW/L) by using a spectrophotometer (Shimazu, Japan). The culture was diluted to the linear range with 0.15 M NaCl. To analyze the extracellular metabolites, such as succinate, acetate, lactate, ethanol, formate, and pyruvate, as well as the residual carbon sources glucose and xylose, 1 mL of the culture was centrifuged at 12 000 rpm for 2 min; the supernatant was then filtrated through a 0.22-μm syringe filter and quantitatively examined by using high-performance liquid chromatography (HPLC) (Shimadzu, Japan) equipped with a refractive index detector (RID-10A) (Shimadzu, Japan) and an Aminex HPX-87H ion exclusion column (Bio-Rad, USA). A 5 mM H_2_SO_4_ solution was then used as a mobile phase at a flow rate of 0.6 mL/min to the column at 65 °C. Standards were prepared for glucose, xylose, pyruvate, succinate, lactate, formate, acetate, and ethanol, and calibration curves were created. The detection sensitivity was 0.1 μg compounds per HPLC assay (10 μL). The detection limit for the extracellular metabolites and carbon sources was 10 mg/L. PHB was quantitatively analyzed using gas chromatography^45^. Briefly, liquid cultures were centrifuged at 10,000 g for 10 min, and then the cells were washed twice in saline and lyophilized overnight. About 3–5 mg lyophilized cell mass was mixed with 1 ml chloroform and 1 ml methanol containing 15% (v/v) sulfuric acid. The methanolysis was performed at 100 °C for 1–3 h in an oil bath. Then 0.5 ml water was added to the mixture and mixed thoroughly for 20 s. After phase separation, the heavier chloroform phase was transferred to another new vial for GC analysis. The PHB content was defined as the percentage ratio of the PHB concentration to biomass.

### Calculation of kinetic parameters

Glucose consumption (*q*_Glc_), and xylose consumption (*q*_Xyl_) were determined for characterizing the strains. These rates were calculated using linear regression during the exponential growth phase. The linear least squares fit to the data resulted in *R*^*2*^ values equal to or higher than 0.97.

### Statistical analysis

Statistical analyses were performed by using SPSS for Windows (version 13.0). A *P* value of <0.05 was considered statistically significant.

## Additional Information

**How to cite this article**: Liang, Q. *et al.* Comparison of individual component deletions in a glucose-specific phosphotransferase system revealed their different applications. *Sci. Rep.* **5**, 13200; doi: 10.1038/srep13200 (2015).

## Supplementary Material

Supplementary Information

## Figures and Tables

**Table 1:** Strains used in this study.

**Strains**	**Relevant characteristics**	**Source**
W3110	*F*^*−*^, *λ*^*−*^, *rph-1*, *IN*(*rrnD, rrnE*)	Lab stock
W3110I	W3110(Δ*ptsI*::FRT)	This study
W3110H	W3110(Δ*ptsH*::FRT)	This study
W3110C	W3110(Δ*crr*::FRT)	This study
W3110G	W3110(Δ*ptsG*::FRT)	This study
YL104	MG1655(Δ*ptsG*Δ*poxB*Δ*pta*Δ*iclR*Δ*sdhAΔarcAΔadhE ldhA::trc-*rbs*-glf*_*ZM*_)	(Li *et al.*, 2013)
YL104H	YL104(Δ*ptsH*::FRT)	This study

**Table 2:** Co-utilization characteristics of the mutants in a mixture of glucose and xylose.

**Strains**	**Sugar ratio**^a^	***q*_(Glc+Xyl)_(g/g CDW/h)**	***q*_Glc_(g/g CDW/h)**	***q*_Xyl_(g/g CDW/h)**	***q*_Glc_/*q*_Xyl_**
W3110	Glc:Xyl = 1:1	N/A	1.05	0.56	1.88
W3110I	Glc:Xyl = 1:1	1.12	0.24	0.88	0.27
W3110H	Glc:Xyl = 1:1	0.53	0.13	0.40	0.33
W3110C	Glc:Xyl = 1:1	1.12	0.72	0.40	1.80
W3110G	Glc:Xyl = 1:1	0.75	0.37	0.38	0.97

^a^The ratio indicated the initial mass concentration ratio of glucose (Glc) to xylose (Xyl) in the medium.

**Table 3:** Succinate yield comparison of the engineering *E. coli* strains.

**Strains and**	**Biomass**	**Glucose (g/L)**		**Succinate (mM)**		**Yield (mol/mol)**			**Productivity (g/L/h)**
**Sugar ratio**	**(g CDW/L)**	**Aerobic**	**Anaerobic**	**Aerobic**	**Anaerobic**	**Aerobic**	**Anaerobic**	**Overall**	**Overall**
YL104	1.93	18.21	10.28	79.15	83.47	0.78	1.46	1.03	0.27
YL104H	2.73	20.93	13.75	104.66	126.02	0.90	1.65	1.20	0.39
