# Supplementary material for: Comparison of individual component deletions in a glucose-specific phosphotransferase system revealed their different applications
Source: Sci Rep. 2015 Aug 19;5:13200. doi: 10.1038/srep13200 (PMC4541071; doi:10.1038/srep13200)
Supplement: Supplementary Information [file srep13200-s1.doc]

**Comparison of individual component deletions in a glucose-specific phosphotransferase system revealed their different applications**

Supplementary Tables

Quanfeng Liang, Fengyu Zhang, Xu Zhang, Yikui Li, Jiaojiao Li, Qingsheng Qi*

State Key Laboratory of Microbial Technology, Shandong University, Jinan 250100, P. R. China

* Corresponding author: Tel & Fax: +86-531-88365628;

E-mail address: [qiqingsheng@sdu.edu.cn](mailto:qiqingsheng@sdu.edu.cn).

**Supplementary Table S1.** Oligonucleotides used in this study.

**Supplementary Table S2.** Substrate co-utilization characteristics of the mutants on different ratio of glucose and xylose.

**Supplementary Table S1.** Oligonucleotides used in this study.

| Oligonucleotides | Sequence |
| --- | --- |
| Gene knockout |  |
| *ptsI*-pKD4-F | 5’-AGATAAGCACATGACAGCTGACGCAGCTGCTCATGAAGTGTGTAGGCTGGAGCTGCTTC-3’ |
| *ptsI*-pKD4-R | 5’-GTGTACTGCGTTAAATCATTGGTGCCGATACTAAAGAAAATGGGAATTAGCCATGGTCC-3’ |
| *ptsH*-pKD4-F | 5’-GGCTTCACTTCTGAAATTACTGTGACTTCCAACGGCAAAGTGTAGGCTGGAGCTGCTTC-3’ |
| *ptsH*-pKD4-R | 5’-GGTACCTTGAGTCAGGCCCAGAGTCTGCAGTTTAAACAGATGGGAATTAGCCATGGTCC-3’ |
| *crr*-pKD4-F | 5’-TTTTTGCGGAAAAAATCGTTGGTGATGGTATTGCTATCAGTGTAGGCTGGAGCTGCTTC-3’ |
| *crr*-pKD4-R | 5’-GCGGCAGATCAAATTCAATGACAGTATCGCCAACTTTCAATGGGAATTAGCCATGGTCC-3’ |
| *ptsG*-pKD3-F | 5’-ACGTAAAAAAAGCACCCATACTCAGGAGCACTCTCAATTGTGTAGGCTGGAGCTGCTTC-3’ |
| *ptsG*-pKD3-R | 5’-AGCCATCTGGCTGCCTTAGTCTCCCCAACGTCTTACGGAATGGGAATTAGCCATGGTCC-3’ |
| Gene test |  |
| *ptsI*-test-F | 5’-ATGATTTCAGGCATTTTAGC-3’ |
| *ptsI*-test-R | 5’-TTAGCAGATTGTTTTTTCTT-3’ |
| *ptsH*-test-F | 5’-ATGTTCCAGCAAGAAGTTAC-3’ |
| *ptsH*-test-R | 5’-TTACTCGAGTTCCGCCATCA-3’ |
| *crr*-test-F | 5’-ATGGGTTTGTTCGATAAACT-3’ |
| *crr*-test-R | 5’-TTACTTCTTGATGCGGATAA-3’ |
| *ptsG*-test-F | 5’-GGAAGAACTGGCGCAGGTAA-3’ |
| *ptsG*-test-R | 5’-CCCCAACGTGGAAGGTTCTAT-3’ |

The letters with underline represent homologous sequences for recombination.

**Supplementary Table S2.** Substrate co-utilization characteristics of the mutants on different ratio of glucose and xylose.

| Mutant | Sugar ratio^a^ | *q*_(Glc+Xyl)_^b^  (g g CDW^-1^ h^-1^) | *q*_Glc_^c^  (g g CDW^-1^ h^-1^) | *q*_Xyl_^c^  (g g CDW^-1^ h^-1^) | *q*_Glc_/*q*_Xyl_ |
| --- | --- | --- | --- | --- | --- |
| W3110I | Glc:Xyl=3:2 | 1.15 | 0.15 | 1.01 | 0.15 |
| W3110H | Glc:Xyl=3:2 | 0.75 | 0.25 | 0.51 | 0.49 |
| W3110C | Glc:Xyl=3:2 | 1.54 | 0.99 | 0.55 | 1.83 |
| W3110G | Glc:Xyl=3:2 | 1.04 | 0.25 | 0.80 | 0.31 |
| W3110I | Glc:Xyl=2:3 | 2.67 | 0.36 | 2.32 | 0.15 |
| W3110H | Glc:Xyl=2:3 | 1.70 | 0.66 | 1.04 | 0.64 |
| W3110C | Glc:Xyl=2:3 | 2.88 | 1.45 | 1.42 | 1.02 |
| W3110G | Glc:Xyl=2:3 | 1.86 | 0.30 | 1.56 | 0.19 |

^a^ Ratio indicated the mass concentration ratio of glucose (Glc) to xylose (Xyl) in the medium.

^b^ The maximum specific consumption rates of total substrates (*q*_(Glc+Xyl)_) were calculated using linear regression during the exponential growth phase. The linear least squares fit to the data displayed *R^2^* values equal or higher than 0.97.

^c^ The consumption rates of glucose (*q*_Glc_) and xylose consumption (*q*_Xyl_) were calculated by the percent of the specified sugar of the total consumed substrates during the exponential growth phase multiplying the maximum specific consumption rates of total substrates (*q*_(Glc+Xyl)_).
